# Molecular characterization and protective efficacy of a new conserved hypothetical protein of *Eimeria tenella*

**DOI:** 10.1051/parasite/2021037

**Published:** 2021-05-03

**Authors:** Huanzhi Zhao, Shunhai Zhu, Qiping Zhao, Bing Huang, Guiling Liu, Zhihang Li, Lu Wang, Hui Dong, Hongyu Han

**Affiliations:** 1 Key Laboratory of Animal Parasitology of Ministry of Agriculture, Shanghai Veterinary Research Institute, CAAS 200241 Shanghai PR China; 2 College of Life and Environment Sciences, Shanghai Normal University 200234 Shanghai PR China

**Keywords:** *Eimeria tenella*, Conserved hypothetical protein, Characterization, Vaccine, Chicken coccidiosis

## Abstract

*Eimeria tenella* is an obligate intracellular parasite that actively invades cecal epithelial cells of chickens. This parasite encodes a genome of more than 8000 genes. However, more than 70% of the gene models for this species are currently annotated as hypothetical proteins. In this study, a conserved hypothetical protein gene of *E. tenella*, designated *Et*CHP18905, was cloned and identified, and its immune protective effects were evaluated. The open reading frame of *Et*CHP18905 was 1053bp and encoded a protein of 350 amino acids with a molecular weight of 38.7kDa. The recombinant *Et*CHP18905 protein (r*Et*CHP18905) was expressed in *E. coli*. Using western blot, the recombinant protein was successfully recognized by anti GST-Tag monoclonal antibody and anti-sporozoites protein rabbit serum. Real-time quantitative PCR analysis revealed that the *Et*CHP18905 mRNA levels were higher in sporozoites than in unsporulated oocysts, sporulated oocysts and second-generation merozoites. Western blot analysis showed that *Et*CHP18905 protein expression levels were lower in sporozoites than in other stages. Immunofluorescence analysis indicated that the *Et*CHP18905 protein was located on the surface of sporozoites and second-generation merozoites. Inhibition experiments showed that the ability of sporozoites to invade host cells was significantly decreased after treatment with the anti-r*Et*CHP18905 polyclonal antibody. Vaccination with r*Et*CHP18905 protein was able to significantly decrease mean lesion scores and oocyst outputs as compared to non-vaccinated controls. The results suggest that the r*Et*CHP18905 protein can induce partial immune protection against infection with *E. tenella* and could be an effective candidate for the development of new vaccines.

## Abstract (fr)

*Eimeria tenella* est un parasite intracellulaire obligatoire qui envahit activement les cellules épithéliales du caecum des poulets. Ce parasite code un génome de plus de 8000gènes. Cependant, plus de 70% des modèles de gènes de cette espèce sont actuellement annotés en tant que protéines hypothétiques. Dans cette étude, un gène de protéine hypothétique conservé d’*E. tenella*, désigné par *Et*CHP18905, a été cloné et identifié, et ses effets immuno-protecteurs ont été évalués. Le cadre de lecture ouvert d’*Et*CHP18905 était de 1053 pb et codait pour une protéine de 350 acides aminés avec un poids moléculaire de 38,7kDa. La protéine recombinante *Et*CHP18905 (r*Et*CHP18905) a été exprimée dans *E. coli*. En utilisant le Western blot, la protéine recombinante a été reconnue avec succès par un anticorps monoclonal anti-GST-Tag et un sérum de lapin anti-protéines de sporozoïtes. Une analyse PCR quantitative en temps réel a révélé que les niveaux d’ARNm d’*Et*CHP18905 étaient plus élevés dans les sporozoïtes que dans les oocystes non sporulés, les oocystes sporulés et les mérozoïtes de deuxième génération. L’analyse par Western blot a montré que les niveaux d’expression de la protéine *Et*CHP18905 étaient plus faibles dans les sporozoïtes que dans les autres stades. L’analyse par immunofluorescence a indiqué que la protéine *Et*CHP18905 était localisée à la surface des sporozoïtes et des mérozoïtes de deuxième génération. Des expériences d’inhibition ont montré que la capacité des sporozoïtes à envahir les cellules hôtes était significativement diminuée après le traitement par l’anticorps polyclonal anti-r*Et*CHP18905. La vaccination avec la protéine r*Et*CHP18905 a permis de réduire significativement les scores moyens des lésions et les sorties d’oocystes par rapport aux témoins non vaccinés. Les résultats suggèrent que la protéine r*Et*CHP18905 peut induire une protection immunitaire partielle contre l’infection par *E. tenella* et pourrait être un candidat efficace pour le développement de nouveaux vaccins.

## Introduction

Avian coccidiosis in the poultry industry is a common disease caused by obligate apicomplexan parasites of the genus *Eimeria*. At present, coccidiosis has historically been controlled by anticoccidial drugs and live vaccines [34]. However, long-term prophylactic drug usage has promoted drug-resistance. As pressure to reduce drug use in poultry production intensiﬁes industry-wide, novel vaccination strategies are needed. Hence, new strategies such as DNA vaccines and subunit vaccines have received widespread attention. Therefore, it is urgent to find novel immunoprotective antigens.

*Eimeria tenella* is one of the 7 recognized species of *Eimeria* that infect chickens. More than 8000 genes of *E. tenella* have been identified throughout the genome [30]. Significant progress has been achieved over the past few several years identifying *E. tenella* genes involved in development, differentiation, virulence, and susceptibility to therapy. However, the identification of most genes in the genome remains unknown [2, 30]. The genome of the Houghton strain of *E. tenella* has been sequenced [30]. The data show that more than 70% of gene models are currently annotated as hypothetical proteins in *E. tenella* [2]. These conserved proteins may be important for invasion, development or the *E. tenella* life cycle. In 2016, Zhai et al. characterized the conserved protein *Et*CHP559 and studied the function and immunogenicity of *Et*CHP559 [47]. However, there are many conserved proteins that have neither been studied nor tested for their function.

In the present study, a new conserved hypothetical protein of *E. tenella, Et*CHP18905 (NCBI reference sequence accession number: XP_013231819), was cloned and recombinant protein GST-*Et*CHP18905 (r*Et*CHP18905) was expressed in an *Escherichia coli* BL21 (DE3) expression system. Polyclonal anti-r*Et*CHP18905 antibodies were generated and used to localize *Et*CHP18905 in parasites by immunofluorescence and to assess inhibitory effects in an *in vitro* assay. The results of the present study indicate that *Et*CHP18905 may participate in parasite invasion, growth and development.

## Materials and methods

### Ethics considerations

All experiments involving animals were approved by the Institutional Animal Care and Use Committee of Shanghai Veterinary Research Institute, the Chinese Academy of Agricultural Sciences (approval no. SHVRI-SZ-20180106-3), and were conducted in strict compliance following the recommendations outlined in the Guide for the Care and Use of Laboratory Animals.

### Animals, parasites and cells

One-day-old Chinese Pudong yellow broilers were obtained from Shanghai Fuji Biological Technology Co., Ltd and reared in steel cages with a wire floor. Animals were provided with water and feed *ad libitum*. The birds were placed in a coccidia-free environment. BALB/c mice were purchased from Shanghai Lingchang Biological Technology Co., Ltd. New Zealand rabbits were obtained from Shanghai SLAC Laboratory Animal Co. Ltd.

*Eimeria tenella* (CAAS21111601) was obtained from the Shanghai Veterinary Research Institute, Chinese Academy of Agricultural Sciences. The parasites were propagated by inoculating 2-week-old chickens, as previously described [40]. Unsporulated (UO) and sporulated oocysts (SO) were obtained and purified using standard procedures [35]. Sporozoites (Spz) were purified *in vitro* from cleaned SO [28]. Second-generation merozoites (Mrz) were collected from the cecal mucosa scraped from the cecum and the cecal contents of chickens at 115h post inoculation (p.i.) and then purified with Percoll [35].

The chicken fibroblast cell line DF-1 (ATCC CRL-12203) was used for *in vitro* inhibition and immunofluorescence assays [19].

### Cloning and sequence analysis of *Et*CHP18905

Total RNA was extracted from Spz using TRIzol reagent (TaKaRa, Tokyo, Japan) according to the manufacturer’s protocol. Total RNA was extracted from 2.0×10^7^ Spz, counted with a hemocytometer using TRIzol reagent (TaKaRa), according to the manufacturer’s protocol. RNA samples were resuspended in diethylpyrocarbonate (DEPC) treated water. Complementary DNA (cDNA) was generated from the total RNA with oligo dT primer and SuperScript™ III Reverse Transcriptase (Invitrogen, Carlsbad, CA, USA).

The complete coding region of *Et*CHP18905 (GenBank accession number: XP_013231819) was amplified by PCR. *Et*CHP18905-specific primers (forward primer: 5′–GATGGACCGAGACCGTCGCTC–3′; reverse primer: 5′–GCGCTGTGGGGGCTCGGGTCG–3′) were used for the PCR assays with the cDNA of Spz as a template. The amplification products were analyzed by 1% agarose gel electrophoresis and purified using a QIAquick^®^ Gel Extraction Kit (QIAGEN, Düsseldorf, Germany). The *Et*CHP18905 fragment was subcloned into the pGEM-T-Easy Vector (Promega, Madison, WI, USA) using T4 DNA ligase to construct a recombinant plasmid pGEM-T-*Et*CHP18905. The recombinant plasmid was subjected to DNA sequencing by Sangon (Shanghai, China).

The full-length cDNA sequence was analyzed using a BLAST search in GenBank (http://www.ncbi.nlm.nih.gov/BLAST/) and the *E. tenella* genome database (http://www.genedb.org/Homepage/Etenella). The molecular mass and theoretical isoelectric point were predicted using the ProtParam tool at the ExPASy server (http://web.expasy.org/protparam/). Signal peptides, transmembrane motifs, and protein motifs were predicted using the computational tools SignalP (http://www.cbs.dtu.dk/services/SignalP/), TMHMM (http://www.cbs.dtu.dk/services/TMHMM-2.0/), and Motif Scan (http://hits.isb-sib.ch/cgi-bin/motif_scan), respectively.

### Expression and puriﬁcation of the recombinant *Et*CHP18905 protein

A 1053-bp fragment of *Et*CHP18905 was amplified from the plasmid pGEM-T-*Et*CHP18905 with the primers: forward primer, 5′–GGAATTCATGGACCGAGACCGTCGCTC–3′; reverse primer, 5′–GCGTCGACGCTGTGGGGGCTCGGGTCG–3′, and ligated into the pGEX-4T-1 vector at the *EcoR*I and *Sal*I cloning sites (underlined). The recombinant pGEX-4T-*Et*CHP18905 plasmid was transformed into *E. coli* BL21 (DE3) cells (Tiangen, Beijing, China). r*Et*CHP18905 expression in *E. coli* was induced by addition of 0.8mM Isopropyl-β-D-1-thiogalactopyranoside (IPTG; Sigma, St Louis, MO, USA) to the bacteria culture after the OD_600_ of the culture reached 0.6at 37°C. The bacteria were collected by centrifugation at 8000×*g* for 10minat 4°C. The bacteria were lysed by sonication and then the bacteria lysates were analyzed by 12% (w/v) sodium dodecyl sulfate polyacrylamide gel electrophoresis (SDS-PAGE). The r*Et*CHP18905 protein was purified by cut SDS polyacrylamide gel [3]. The concentration of the sample was determined using a BCA protein assay kit (Beyotime, Haimen, China). The protein was stored at −20°C for later analysis.

### Generation of anti-r*Et*CHP18905 and anti-sporozoite polyclonal serum

The Spz proteins were prepared using sonication as described by Jiang et al. [19]. To generate polyclonal antibodies, either 50μg or 200μg of the puriﬁed r*Et*CHP18905 protein or the Spz protein was mixed with Freund’s complete adjuvant (Sigma) in a 1:1 mixture and injected into six-week-old BALB/c mice or two-month-old New Zealand white rabbits, respectively. After two weeks, mice and rabbits were immunized with the same dose of antigen emulsified with Freund’s incomplete adjuvant (Sigma). And then, the rabbits and mice were re-boosted four times at intervals of 1 week. Finally, the polyclonal antibody serum was collected and stored at −80°C until use. Pre-immune serum was collected from the rabbits’ ear vein before immunization for further use as the negative control.

### Western blot analysis for r*Et*CHP18905

In order to verify the expression of r*Et*CHP18905, the puriﬁed r*Et*CHP18905 protein was separated by 12% SDS-PAGE. All separated proteins were electrically transferred onto polyvinylidene fluoride (PVDF) membrane (Merck Millipore, Billerica, MA, USA). The membranes were blocked in phosphate-buffered saline (PBS) containing 5% skimmed milk, washed with PBS containing 0.05% Tween 20 (PBS-T, pH 7.2), and incubated overnight at 4°C with the anti GST monoclonal antibody (1:2000) (CoWin Biosciences, Beijing, China) and rabbit anti-Spz proteins of *E. tenella* serum (1:200), respectively. Secondary antibodies, IRDye^®^ 800CW Donkey anti-Mouse IgG and IRDye^®^ 800CW Goat anti-Rabbit IgG (1:10,000) (LI-COR, Lincoln, NE, USA) were then applied at 37°C for 1h. Naïve rabbit serum (1:200) was used as the negative control. Membranes were scanned with an Odyssey^®^ Infrared Imaging System (LI-COR).

### *Et*CHP18905 transcript levels in different developmental stages of *E. tenella*

Transcription profiles of *Et*CHP18905at different developmental stages of *E. tenella* (UO, SO, Spz, and Mrz) were determined using real-time quantitative PCR (qPCR). Total RNAs were isolated by TRIzol reagent (Invitrogen) from UO, SO, Spz, and Mrz. RNA preparations were treated with RNase-free DNase Ι (Invitrogen) to remove DNA contamination. In brief, 1.0×10^7^ purified UO or SO were oscillated and broken in 500μL of TRIzol with equal volume of 710–1180μm glass beads (Sigma) for 10min (4000rpm). A total of 2.0×10^7^ Spz or Mrz were lysed in 500μL of TRIzol. Total RNAs were precipitated with isopropanol and washed with 75% ethanol and then resuspended in DEPC treated water. cDNA was synthesized with SuperScript II reverse transcriptase (Invitrogen) and random primers (Invitrogen). qPCR was performed with SYBR1 Green I dye (Takara) on a StepOne™ Real-Time PCR System (Thermo Fisher Scientific, Waltham, MA, USA). qPCR primers for *Et*CHP18905 were: 5′–TCCCCTCAAGCCCCTCATACAGT–3′ (forward) and 5′–CCAGCACTAAGTCCACTGAACGC–3′ (reverse). A housekeeping gene of *E. tenella*, 18S ribosomal RNA, was used as the reference gene and was amplified using the primers 5′–TGTAGTGGAGTCTTGGTGATTC–3′ (forward) and 5′–CCTGCTGCCTTCCTTAGATG–3′ (reverse). The reactions for each sample were performed in triplicate, and the experiment was repeated three times. The transcription levels were quantified with the 2^−ΔΔ*Ct*^ method [27].

### *Et*CHP18905 protein expression in four development stages of *E. tenella*

Total proteins were prepared from four life cycle stages of *E. tenella* using a commercially available cell-lysis buffer for western blot and immunoprecipitation (Beyotime). Protein concentrations were determined with a BCA protein assay kit (Beyotime). The protein lysate from each sample was separated by SDS-PAGE and transferred to PVDF membrane (Merck Millipore). Membranes were blocked for 2h with 5% (w/v) skimmed milk powder in PBS, followed by incubation with mouse polyclonal anti-r*Et*CHP18905 (1:100) and mouse monoclonal anti-α-tubulin (1:5000) at 37°C for 2h, respectively. Secondary antibodies, HRP-conjugated Affinipure Goat anti-Mouse IgG (H+L) (1:5000) (Proteintech, Rosemont, IL, USA) were incubated at room temperature for 45min, and bands were detected using ChemiDoc (Bio-Rad, Hercules, CA, USA). α-tubulin (Sigma) was used as an internal reference for protein extracts at each stage. For comparative quantitative protein expression profile analysis, the resulting images were analyzed by Image J (Rawak Software Inc., Stuttgart, Germany) software.

### Localization of *Et*CHP18905 by indirect immunofluorescence

The location of *Et*CHP18905 in Spz, Mrz and parasites invaded DF-1 cells were assessed by indirect immunofluorescence assay (IFA) with anti-r*Et*CHP18905, as previously described [19]. Six-well plates (Corning Inc., Corning NY, USA) precoated with coverslips were seeded with DF-1 cells (2×10^5^ cells per well). These cells were sequentially cultured in complete medium (CM, DMEM with 10% fetal bovine serum and 100 units/mL penicillin/streptomycin) at 37°C and 5% CO_2_ for 24h. Freshly cleaned Spz (6×10^5^ parasites per well) were added to invade and develop in the DF-1 cells. The coverslips were collected and washed at 2, 48 and 72h p.i., respectively. Subsequently, all the coverslips were fixed in 4% paraformaldehyde for 20min, permeabilized with 1% Triton X-100 in PBS for 15min, and then blocked with 2% bovine serum albumin in PBS overnight at 4°C. After washing four times, rabbit anti-r*Et*CHP18905 polyclonal antibody (1:100 dilution) with PBS was added for incubation at 37°C for 2h. Then the goat anti-rabbit IgG fluorescein isothiocyanate (FITC)-conjugated antibody (1:500 dilution) (Sigma) was added, and the samples were incubated at 37°C for 1h. Nuclei of parasites and cells were stained with 15μg/mL 4, 6-diamidino-2-phenylindole (DAPI) (Beyotime) for 30minat room temperature. At the end of each step, all the coverslips were washed three times in PBS. The coverslips were placed on glass slides using 60μL of Fluoromount Aqueous Mounting Medium (Sigma) and observed by a laser scanning confocal microscope (Zeiss LSM800 microscope, Carl Zeiss, Germany). Spz were incubated in PBS or CM for 2hat 41°C, and air dried on coverslips before fixation. Mrz were also incubated in PBS for observation. Spz or Mrz were evenly smeared onto glass coverslips to localize the protein in Spz and Mrz. After air drying, the coverslips were prepared for immunofluorescence using the method described above.

### Invasion inhibition assays

Invasion inhibition assays were performed to investigate whether anti-r*Et*CHP18905 affects Spz invasion of DF-1 cells [19]. Rabbit IgG against r*Et*CHP18905 and GST protein were purified using protein A+G agarose (Beyotime) and the concentration of IgG was determined by a BCA Protein Assay Kit (Beyotime). DF-1 cells (3×10^5^ cells per well) were cultured in flat-bottomed 24-well plates (Corning) in CM at 37°C and 5% CO_2_ for 12h. Freshly cleaned *E. tenella* Spz were labeled for 15min using carboxyfluorescein diacetate succinimidyl ester (CFDA SE) (Invitrogen), according to the manufacturer’s protocol. Then, the labeled Spz were incubated with purified rabbit anti-r*Et*CHP18905 IgG at different concentrations (100, 200, or 300μg/mL) for 2hat 37°C and added to infect DF-1 cells at 41°C, 5% CO_2_ for 12h. Naïve rabbit serum IgG (Sigma, USA) and rabbit anti-GST IgG were used as the negative control and GST control. Labeled Spz incubated with no antibody were the positive control. The cells were washed, trypsinized, harvested, and analyzed on a flow cytometer (model Cytomics FC500; Beckman Coulter, Brea, CA, USA). All assays were performed in triplicate. The inhibition rate was calculated based on the invasion rate and the positive control [17].

### Immunization experimental design

Broilers at 7 days of age were randomly divided into four groups and each group included 12 birds. Broilers were inoculated with a subcutaneous injection of 50μg or 100μg of purified r*Et*CHP18905 protein emulsified in Montanide ISA 71 adjuvant (Seppic, Puteaux, France) in a 3:7 mixture [18]. The challenged and unchallenged control birds were immunized with PBS emulsified in Montanide ISA 71 adjuvant. A booster immunization was given one week later with the same amount of components as the ﬁrst immunization. Subsequently, 7 days after the last immunization, 1×10^4^ SO of *E. tenella* were given to all the birds except for the unchallenged control birds. Unchallenged control chickens were given PBS orally.

### Evaluation of immune protection

The efﬁcacy of immunization was evaluated by the average body weight gain, mean lesion scores, fecal oocyst output, and percentage reduction of oocyst excretion. Body weight was measured on days 0 and 8 post challenge. Fecal samples were collected daily from days 6 to 8 post challenge. Oocysts per gram of fecal sample were counted using a McMaster chamber [9]. The percentage reduction of oocyst excretion was calculated by the formula: (number of oocysts from the challenged-unvaccinated group−number of oocysts from the challenged-vaccinated group)/number of oocysts from the challenged-unvaccinated group×100% [31]. The ceca of each group were collected separately. Intestinal lesions were scored according to the method of Johnson and Reid [20].

### Preparation of the serum

The blood of broilers in each group was collected on day 8 post challenge. For the serum IgG, cytokines, sCD4 and sCD8 determination, the sera were separated from isolated blood samples. In brief, the blood samples were incubated at 37°C for 1h, and centrifuged at 1000×*g* for 5minat 4°C to separate the serum.

### Determination of serum antibody levels

The serum IgG against r*E*tCHP18905 levels were detected by ELISA atday 8 post challenge, as described previously [24]. Briefly, 96-well microtiter plates (Corning) were coated with purified r*Et*CHP18905 (10μg/well) and incubated overnight at 4°C. After three washes with PBS-T, the plate was blocked with PBS containing 1% BSA for 2hat 37°C, and then the plate was washed with PBS-T. The plates were incubated with the serum samples diluted 1:25 in PBS (50μL/well) for 2hat 37°C. After washing five times with PBS-T, the secondary antibodies of HRP-donkey-anti-chicken IgG antibody (50μL/well) (Sigma) (1:5,000 dilution) was added and incubated for 2hat 37°C. The plates were washed five times with PBS-T and developed with 3,3′,5,5′-tetramethylbenzidine. Optical densities at 450nm (OD450) were determined on a microplate spectrophotometer. All assays were performed in triplicate.

### Determination of cytokine, sCD4 and sCD8 levels

The immune stimulation effect of r*Et*CHP18905 protein on broilers was measured by ELISA atday 8 post challenge, as previously described [6, 22, 24, 25]. The levels of cytokines, soluble cluster of differentiation 4 (sCD4), soluble cluster of differentiation 8 (sCD8), interferon-γ (IFN-γ), interleukin-10 (IL-10), interleukin-17 (IL-17), and transforming growth factor (TGF-β1) were determined with “Chicken Cytokine ELISA Quantization Kits” (CUSABIO, Wuhan, China), according to the manufacturer’s instructions.

### Statistical analysis

SPSS version 22 (SPSS, Chicago, IL, USA) was used to analyze body weight gain, mean lesion scores, and fecal oocyst output and oocyst reduction ratio. GraphPad Prism version 6.0 (GraphPad, La Jolla, CA, USA) was used to analyze real-time quantitative PCR (qPCR), invasion inhibition, and antibody and cytokine levels. Differences among groups were analyzed by one-way analysis of variance (ANOVA) and Duncan’s multiple range test (*p*<0.05 was considered significantly different). The lesion scores were compared by the nonparametric Kruskal–Wallis test.

## Results

### Characterization of the *Et*CHP18905 sequence

**Figure 1:**
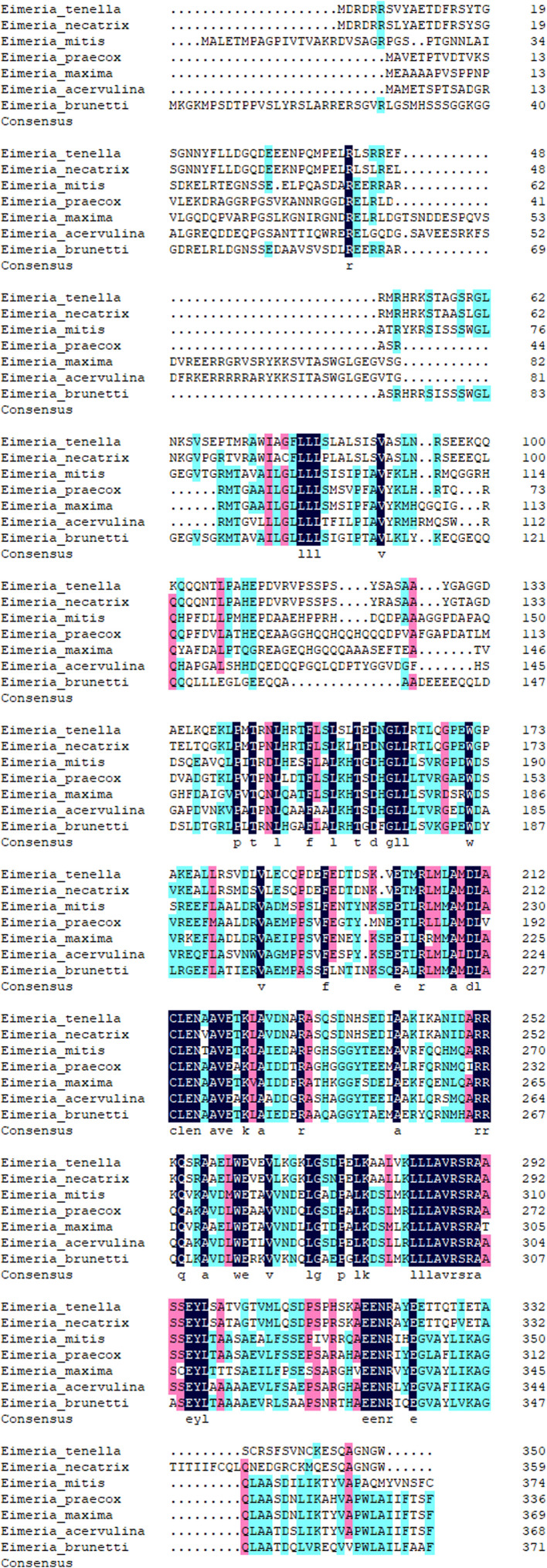
Multiple alignment analysis of *Et*CHP18905 with other *Eimeria* proteins. DNAMAN was used to analyze the deduced protein sequences. The identical amino acids are listed at the bottom. NCBI reference sequence accession numbers: *Eimeria tenella*, XP_013231819, *Eimeria necatrix*, XP_013438465, *Eimeria mitis*, XP_013355934, *Eimeria maxima*, XP_013336337, *Eimeria acervulina*, XP_013251133; GenBank accession numbers: *Eimeria praecox*, CDI76926, *Eimeria brunetti*, CDJ52365.

**Figure 2:**
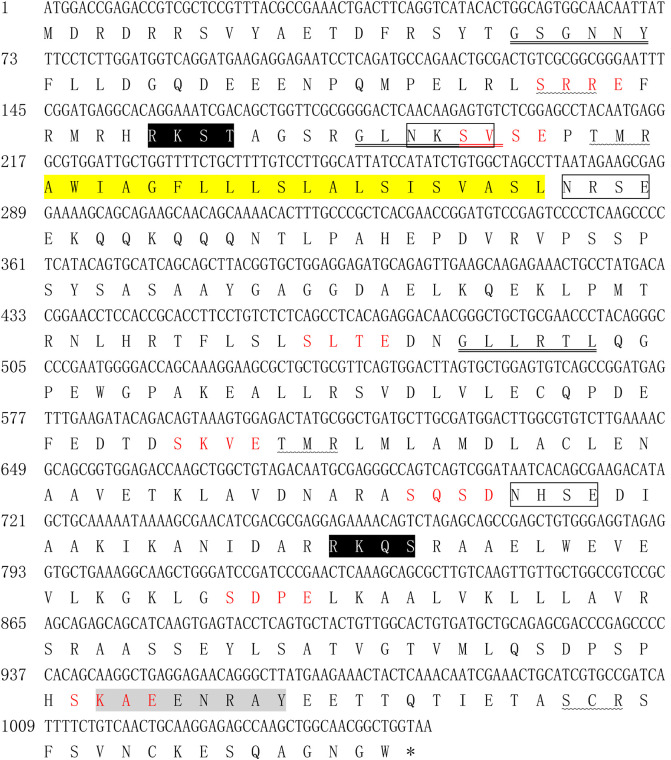
Bioinformatic analysis of *Et*CHP18905. The stop codon is indicated with an asterisk. N-myristoylation sites are double underlined. The transmembrane domain is shaded yellow with black lettering. N-glycosylation sites are surrounded by a black box. cAMP- and cGMP-dependent protein kinase phosphorylation sites are shaded black with white lettering. Tyrosine kinase phosphorylation site is shaded grey with black lettering. Casein kinase II phosphorylation sites are indicated with red lettering. Protein kinase C phosphorylation sites are underlined by a wavy line.

By analysis of the sequence, the open reading frame (ORF) was 1053bp and found to encode a protein of 350 amino acids with a predicted molecular mass of 38.7kDa. Based on BLASTp analysis, the sequence obtained shared 100%, 84.68%, 52.48%, 40.53%, 44.53%, 39.10%, 41.83% and 47.74% amino acid homology with conserved hypothetical protein (CHP) from *E. tenella* (NCBI reference sequence accession number: XP_013231819) and *Eimeria necatrix* (XP_013438465), *Eimeria mitis* (XP_013355934), *Eimeria maxima* (XP_013336337), *Eimeria acervulina* (XP_013251133), *Eimeria praecox* (CDI76926), and *Eimeria brunetti* (CDJ52365) (Fig. 1), respectively. Analysis of the amino acid sequence showed that a transmembrane domain (amino acid sequence 73–92) was found in the deduced protein, but no signal peptide was detected. Structural module and conservative structure predictions indicated that the protein contains seven casein kinase II phosphorylation sites, four protein kinase C sites, three N-glycosylation sites, three N-myristoylation sites, two cAMP and cGMP-dependent protein kinases phosphorylation sites and a tyrosine kinase phosphorylation site (Fig. 2).

### Expression and purification of recombinant *Et*CHP18905

**Figure 3:**
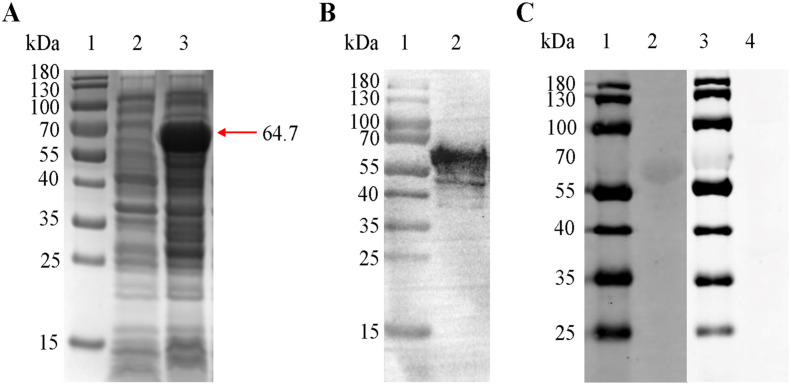
Expression and purification of r*Et*CHP18905. (A) SDS-PAGE analysis of the *5*r*Et*CHP18905. Lanes 1, protein marker; 2, negative control (not induced with IPTG); 3, the r*Et*CHP18905 protein with the GST-tag protein of the vector (induced with IPTG for 6h). (B) Western blot analysis of purified r*Et*CHP18905 protein. Lane 2, protein recognized by an anti GST-Tag monoclonal antibody. (C) Western blot analysis of purified r*Et*CHP18905 protein. Lane 2, protein recognized by rabbit sera against sporozoite, lane 4 incubated with naïve rabbit serum.

SDS-PAGE analysis showed that the recombinant protein was expressed in *E. coli* BL21 cells successfully and found mainly in the precipitate. After purification by cut SDS polyacrylamide gel, the expected protein band of 64.7kDa (including with GST tag) (Fig. 3A) was observed in the SDS-acrylamide gel. Western blot analysis indicated that the recombinant protein was recognized by the anti-GST monoclonal antibody (Fig. 3B) and rabbit sera against Spz of *E. tenella* (Fig. 3C, lane 2). Normal rabbit serum failed to detect any protein of the expected molecular weight of r*Et*CHP18905 (Fig. 3C, lane 4).

### Transcription of *Et*CHP18905at different developmental stages of *E. tenella*

**Figure 4:**
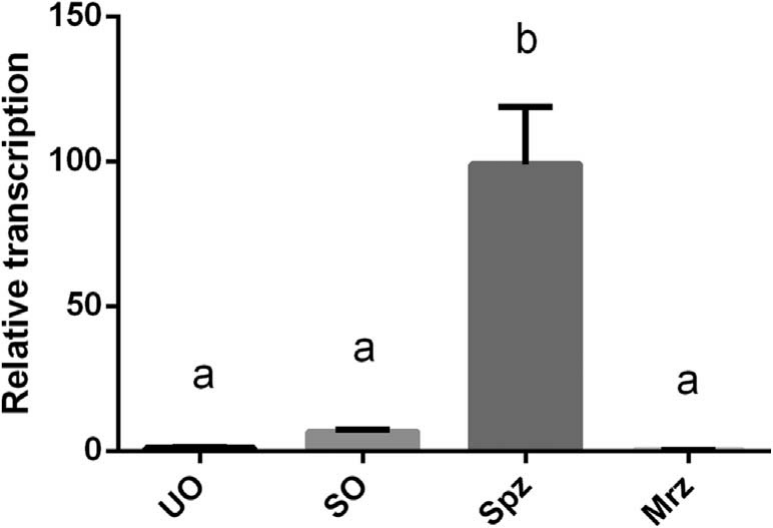
Transcription levels of *Et*CHP18905 in different developmental stages of *E. tenella*. UO, unsporulated oocysts; SO, sporulated oocysts; Spz, sporozoites; Mrz, merozoites. Bars with different letters indicate significantly different expression levels (*p*<0.05) and the error bars indicate standard deviations.

qPCR results showed that the levels of *Et*CHP18905 mRNA in UO, SO and Mrz were similar (*p*>0.05), which were significantly lower than those in the Spz (*p*<0.05) (Fig. 4).

### *Et*CHP18905 protein expression level in *E. tenella*

**Figure 5:**
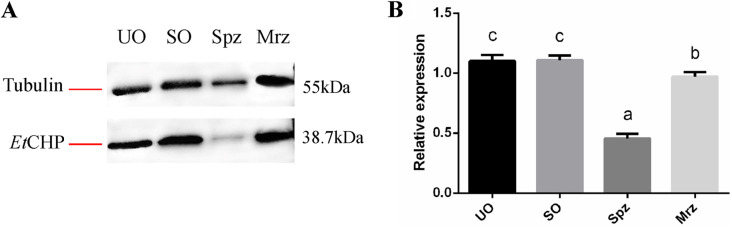
Expression levels of *Et*CHP18905 in different developmental stages of *E. tenella*. (A) Western blot of the internal reference tubulin and *Et*CHP18905 protein. (B) Relative expression levels of the *Et*CHP18905 protein. Bars with different letters indicate significantly different expression levels (*p*<0.05) and the error bars indicate standard deviations.

Western blot analysis results indicated that *Et*CHP18905 expression levels were higher in the UO and SO than Mrz and Spz stages, and *Et*CHP18905 expression levels were lowest in the Spz stage (Fig. 5).

### Localization of *Et*CHP18905 during *in vitro* infection

**Figure 6:**
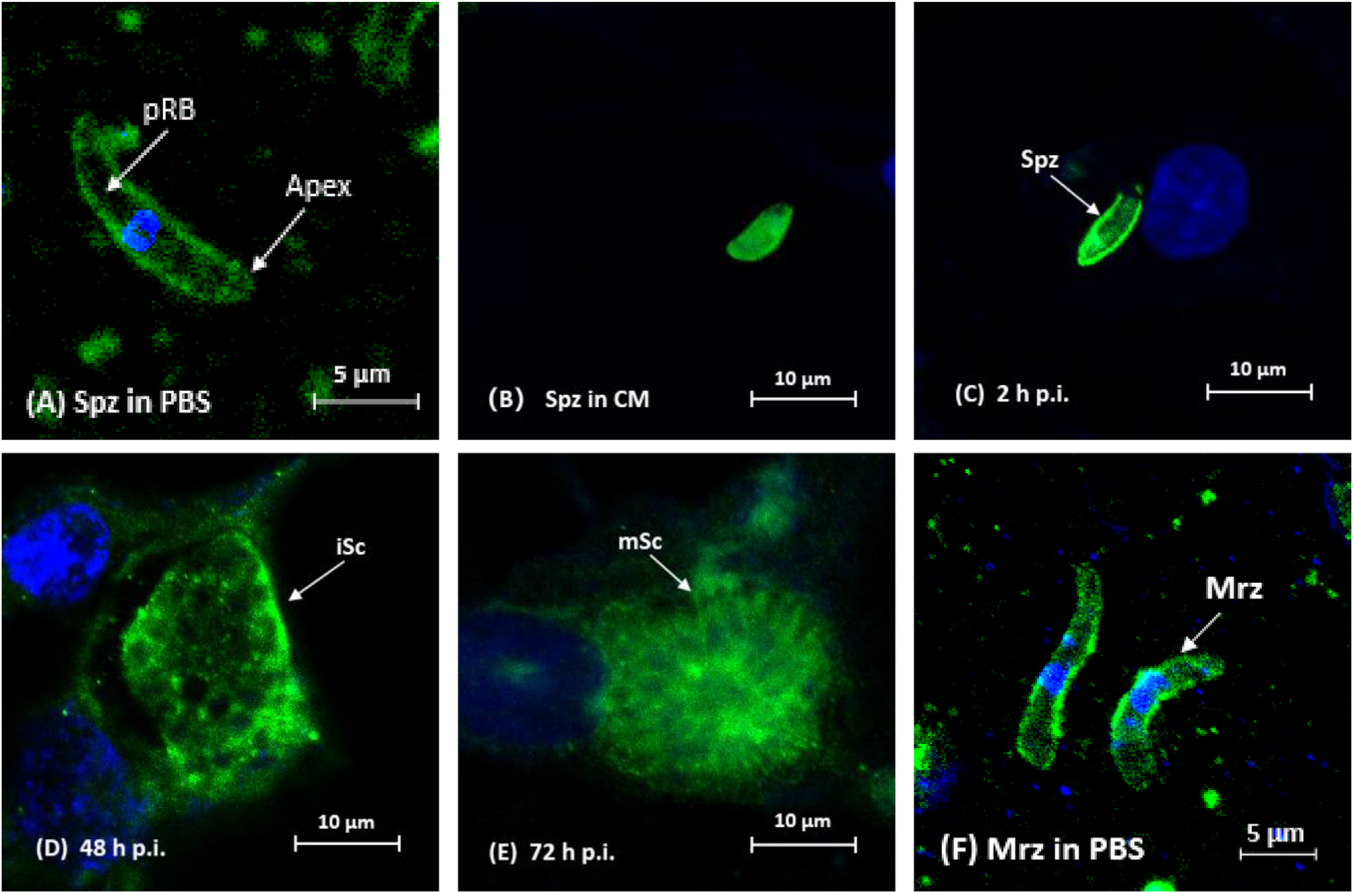
Localization of *Et*CHP18905 in infected DF-1 cells by indirect immunofluorescence. Parasites incubated with anti-r*Et*CHP18905, stained with FITC (green)-conjugated secondary antibodies, and counterstained with DAPI (blue). Infected DF-1 cells were collected at indicated time points post-infection. (A) Sporozoites (Spz) in PBS, pRB, posterior refractile body; (B) Spz in complete medium. Infected DF-1 cells were collected at the indicated time points post-infection (pi); (C) 2hours pi (hpi); (D) immature schizonts (iSC) 48hpi; (E) mature schizonts (mSC) 72hpi; (F) merozoites (Mrz) in PBS.

Indirect immunofluorescence results showed that in Spz incubated with PBS, *Et*CHP18905 was distributed on the surface of Spz (Fig. 6A). *Et*CHP18905 uniformly distributed throughout the cytoplasm in whole Spz after incubation in CM (Fig. 6B). After Spz were added to DF-1 cells for 2h and 48h, *Et*CHP18905 was concentrated on the surface of Spz (Figs. 6C and 6D). After infection for 72h, *Et*CHP18905 was evenly distributed in most areas of the parasite (Fig. 6E). Moreover, *Et*CHP18905 was primarily located on the surface of Mrz (Fig. 6F).

### *In vitro* invasion inhibition assay

**Figure 7:**
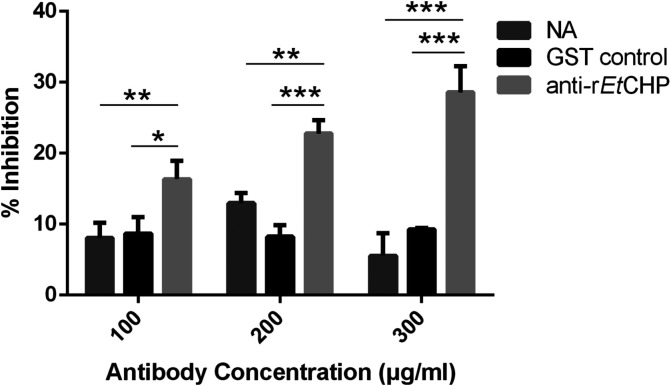
Inhibition of sporozoite invasion *in vitro* by anti-r*Et*CHP18905. Anti-r*Et*CHP18905, rabbit anti-r*Et*CHP18905 IgG; NA, naïve rabbit sera IgG; GST control, rabbit anti-GST IgG. The symbol “*” represents *p*<0.05, “**” represents *p*<0.01, and “***” represents *p*<0.001 for comparison of treatment with anti-r*Et*CHP18905 and naïve rabbit sera IgG and anti-GST IgG at the same concentration. The error bars indicate the standard deviation. All assays were performed in triplicate.

*In vitro* invasion inhibition assay results showed that the inhibition rate was 28% at an antibody concentration of 300μg/mL (Fig. 7). Compared with naïve rabbit IgG and GST control groups, the inhibition effect after pretreatment with anti-r*Et*CHP18905 IgG was significant (*p*<0.01). In contrast, naïve rabbit sera IgG and GST control groups did not have a significant effect on invasion by *E. tenella* Spz.

### Protective efficacy of vaccination on *E. tenella* challenge

Body weight gain, cecal lesion scores, oocyst output and the percentage reduction of oocyst excretion are summarized in Table 1. The results showed that non-immunized challenged control groups exhibited significantly reduced weight gain compared with all immunized groups and the unchallenged control group chickens (*p*<0.05) after challenge. The cecal lesion scores of chickens immunized with r*Et*CHP18905 were significantly lower than those of the challenged control. The oocyst output was reduced after immunization with r*Et*CHP18905. Chickens immunized with r*Et*CHP18905 presented significantly higher percentage reduction of oocyst excretion compared with the challenged controls (*p*<0.05).

**Table 1:** Protective effect of r*Et*CHP protein on *E. tenella* infection.

Group	Average body weight gains (g)	Mean lesion scores	Oocyst shedding per bird (×10^7^)	Percentage reduction of oocyst excretion (%)
Unchallenged control	258.62±70.26c	0.00±0.00a	0.00±0.00a	100d
Challenged control	180.87±45.38a	3.20±0.83c	4.43±0.99c	0.00a
r*Et*CHP-50μg	226.25±24.47b	1.75±0.95b	2.10±0.88b	54.07±11.76c
r*Et*CHP-100μg	235.25±23.44b,c	1.20±0.44b	3.26±2.47b,c	31.99±29.35b

a–d Values with different letters in the same column are significantly different (*P*<0.05) according to the ANOVA Duncan test.

### IgG titers and cytokine, sCD4 and sCD8 concentrations in sera of immunized chickens

**Figure 8:**
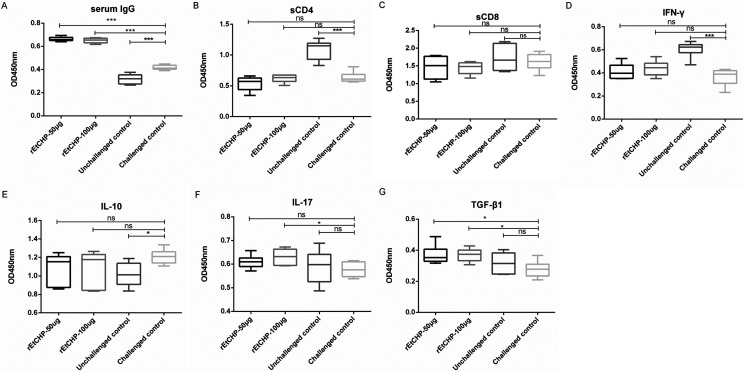
Levels of IgG (A), sCD4 (B), sCD8 (C), cytokines IFN-γ (D), IL-10 (E), IL-17 (F) and TGF-β1 (G) in chicken sera were measured using ELISA. Chickens of group r*Et*CHP18905-50μg and group r*Et*CHP18905-100μg were immunized with 50μg or 100μg of r*Et*CHP18905 protein, respectively. Challenged and unchallenged groups were immunized with PBS and served as controls. The IgG titers and the concentrations of sCD4, sCD8, and cytokines are expressed as Min to Max. (*p<0.05, **p<0.01, ***p<0.001; ns, *p*>0.05).

The results in Figure 8A show that serum from chickens immunized with r*Et*CHP18905 had significantly higher levels of IgG antibody (*p*<0.001) compared with the challenged control group. sCD4 levels in chickens from the two immunized groups were not significantly higher (*p*>0.05) than the challenged control group, but were significantly lower than the unchallenged group (Fig. 8B). No significant differences (*p*>0.05) of sCD8 and IL-10 were observed between the immunized and unimmunized-challenged group (Figs. 8C and 8E). The IFN-γ levels in the 100μg r*Et*CHP18905-immunized group were higher but not significantly than challenged control (Fig. 8D). IL-17 100-μg-immunized group and TGF-β1 in chickens from the two immunized groups all showed significantly higher levels (*p*<0.05) compared with the unchallenged group (Figs. 8F and 8G).

## Discussion

In the present report, a new gene of conserved hypothetical protein from *E. tenella* was cloned and characterized. The 1053bp ORF was shown to encode a 350 amino acid polypeptide of~38.7kDa. Sequence analysis showed that the protein had no signal peptide, and amino acids 73–92 formed a transmembrane region. Given this observation, it is speculated that the protein is anchored to the membrane. Bioinformatics analysis predicted that the protein contains seven tyrosine kinase phosphorylation sites, four protein kinase C sites, three N-glycosylation sites, three N-myristoylation sites, two cAMP and cGMP-dependent proteins kinase phosphorylation sites, and one tyrosine kinase phosphorylation site. These sites and structures indicate that the function of this protein may be regulated by post-translational modifications. The BLASTp results showed that the deduced amino acid sequence of *Et*CHP18905 was 100% homologous to the *E. tenella* conserved hypothetical protein (NCBI reference sequence accession number: XP_013231819, GeneID: ETH_00018905) and 84% homologous to the *E. necatrix* conserved hypothetical protein (NCBI reference sequence accession number: XP_013438465). These results indicate that CHP in *E. tenella* and CHP in *E. necatrix* have high homology. *Eimeria tenella* and *E. necatrix* are the most pathogenic species among the species represented in the genus *Eimeria,* which cause severe tissue damage to the host intestine [33]. In addition, the *Et*CHP18905 protein is a putative interacting protein of the *Et*CDPK3 that we screened by a yeast two-hybrid system. In this series of experiments, although we were not able to show an interaction between *Et*CDPK3 and *Et*CHP using GST pull-down and Co-IP methods (data not shown), we speculated that this protein may be involved in invasion of host cells and development of *E. tenella*.

The mRNA and protein levels of *Et*CHP18905 were examined in four different developmental stages. According to qPCR, the *Et*CHP18905 gene was most prominent in Spz of *E. tenella*, and only weakly detected in UO and Mrz. These results showed that the *Et*CHP18905 gene was transcribed predominantly at a distinct phase of the *E. tenella* life cycle. However, western blot showed that protein levels were weakest for *Et*CHP18905 in SO. A previous study revealed that the ratios between protein and mRNA are mainly determined by translation and protein degradation in a cell [8]. However, the two processes of translation and protein degradation are highly regulated at the overall and gene-specific level [8]. Their study revealed that 15–70% of the variation is explained by posttranscriptional and post-translation regulation and by measurement errors [5]. Hence, this may explain why the mRNA level of *Et*CHP18905 is inconsistent with the protein level. However, the specific reasons for this difference need to be investigated further.

The localization of the *Et*CHP18905 protein in different developmental stages of parasite development was also investigated using an antibody raised against the r*Et*CHP18905. Indirect immunofluorescence showed that *Et*CHP18905 was located on the membrane of *E. tenella* Spz and Mrz stages, which was consistent with the transmembrane protein structure data indicating that the protein was anchored to the membrane. Moreover, immunofluorescence showed that staining was stronger in the Mrz stage. Western blot analysis showed that the expression levels of *Et*CHP18905 in the Mrz stage was higher than in the Spz stage. However, this result was inconsistent with the results at the transcription level. This may be due to post-translational modifications [29]. Results from previous studies have suggested that mRNA abundance is a poor indicator of the levels of the corresponding protein [1, 10, 13]. Furthermore, the expression of *Et*CHP18905 increased after the Spz invaded DF-1 cells for 2h. Thus, this protein might function in Spz invasion or schizonts evolution. The results of the *in vitro* experiments confirm this. *In vitro* invasion inhibition assays using polyclonal antibody against r*Et*CHP18905 showed partial blockage of the invasion of Spz into cells. Inhibition of sporozoites was modest at 28%, at antibody concentrations of 300μg/mL. Likewise, the rate of invasion inhibition increased with the increase of anti-r*Et*CHP18905 IgG concentration. Previous studies have shown that polyclonal antibodies can significantly inhibit Spz invading DF-1 cells [7, 11, 19, 48]. In 2016, Zhai et al. found that rabbit antiserum against r*Et*CHP559 can block invasion of host cells by Spz [47]. The above results suggest that *Et*CHP18905 is related to invasion. This new antigen might be useful for identification of novel vaccine targets, thus improving the knowledge of immunogenic proteins in *E. tenella*.

In previous research, many DNA and recombinant protein vaccines have been reported to induce immuno-protection to live parasite challenge [18, 43, 44]. In the present study, following infection challenge, the body weight gain of non-immunized chickens was reduced significantly compared with immunized chickens. Moreover, chickens immunized with r*Et*CHP18905 had significantly lower lesion scores and fecal oocyst output compared to non-immunized birds. Previous studies of *Eimeria* spp. proteins have shown that a similar effect can be produced after immunization with recombinant protein or recombinant plasmid [25, 26]. The data presented here showed that immunization with r*Et*CHP18905 could produce partial protection against live *E. tenella* infection. However, the difference of oocysts output between challenged control groups and the two immunized groups may be under reality. This was probably due to widespread tissue damage and severe hemorrhage that prevents a large number of merozoites from reinfecting intestinal epithelial cells, resulting in a decrease in average oocyst production [12]. It, therefore, appears likely that the differences in lesion score and oocyst output can be caused by merozoite loss [12]. Furthermore, the decrease in oocyst output is not as high as in previous studies [16, 47]. Thus, the effect of r*Et*CHP18905 in reducing oocyst shedding should be researched further.

Humoral immunity in the immune response against coccidiosis is usually considered to play a minor role [45]. However, as early as 2008, Constantinoiu et al. pointed out that humoral immunity may also contribute to protective immune responses [4]. Their study revealed that chicken infected with an attenuated strain of *E. tenella* mount an antibody response to all lifecycle stages. High levels of antibodies against Spz and Mrz were detected in infected chickens inoculated with SO of *E. tenella* [4]. Moreover, in 1994, Smith also found that antibodies could inhibit parasite development and provide passive immune protection [36]. They found that there was an excellent correlation between antibody titer and protection. Oral infection of hens with *E. maxima* oocysts caused production of antibodies which were passed into the egg yolk and subsequently to hatchlings. The total number of oocysts excreted in the feces of chicks from eggs has decreased after infection with *E. maxima* oocysts [36]. Huang et al. [16] reported that birds immunized with the *E. maxima* rMIC7 protein and pVAX1-MIC7 exhibited higher IgG concentrations than the PBS and pVAX1 controls. In the present study, the IgG concentrations of the r*Et*CHP18905-immunized chickens were significantly higher than the negative controls. These findings confirmed that in rEtCHP18905-immunized chickens, certain humoral immune responses were induced.

Cell-mediated immunity plays a major role against coccidiosis [39]. The concentration of soluble sCD4 and sCD8 in serum is consistent with the number of CD4+and CD8+lymphocytes that produce them [41, 46]. Previous studies reported that levels of sCD4 and sCD8 were higher in experimental groups immunized with r*Em*SAG and r*Em*MIC7 compared with the control [16, 26]. However, the results presented here showed that serum sCD4 and sCD8 were not significantly different in immunized chickens compared to non-immunized controls. This suggests that r*Et*CHP18905 could not stimulate the recruitment of T-cell subpopulations. IFN-γ is reported to be related to protective immune responses to avian coccidiosis [23]. A previous study found that IFN-γ concentrations were higher in *Em*MIC7-vaccinated birds [16]. However, r*Et*CHP18905 increased the serum concentrations of IFN-γ but not significantly compared to the control group. Nevertheless, the relative importance of each cytokine type in inducing immune challenge could not be inferred from these data.

One of the Th2-type cytokines, IL-10 and IL-17 are also involved in immune response to coccidial challenge. IL-10 has been shown to be crucial for control of *Eimeria* infections [32]. Wu et al. observed that serum IL-10 levels increased on day 5 after infection with *E. tenella* [42]. However, in this study, there was no significant level of IL-10 detected in groups immunized with r*Et*CHP18905. Previous reports have shown that the immunization of animals with DNA vaccines produced higher levels of IL-17 [14, 37]. In previous vaccination trials, IL-17 concentrations in the vaccinated groups were significantly higher than those of the unvaccinated groups [16, 26]. In the current study, the concentrations of IL-17 in the immunized groups were also significantly higher than the non-immunized groups. The previous study, and our data, together confirm that IL-17 might be associated with protective immunity to coccidiosis. However, the specific functions of IL-10 and IL-17 in the immunity to coccidiosis requires further investigation.

The Treg-type cytokines TGF-β, produced by Treg cells, have been shown to regulate immunosuppression mechanisms [21]. Two earlier reports showed that TGF-β was significantly higher in chickens immunized with recombinant *Eb*AMA1 [38, 49]. The same result was demonstrated by Liu et al. [26]. Likewise, in the present study, the levels of the TGF-β1 in the r*Et*CHP18905-immunized groups were significantly higher than the non-immunized group. These findings together confirm that TGF-β1 may function in coccidiosis-induced immune pathways.

In summary, Th2-type cytokines can down-regulate the expression levels of Th1-type cytokines and regulate the immune response [15]. In the present study, IL-17 and TGF-β1 levels increased following immunization. In contrast, sCD4, sCD8 and IL-10 did not increase significantly. Moreover, higher IgG concentrations were detected in the *Et*CHP18905 vaccinated chickens. Thus, *Et*CHP18905 might be a supplementary candidate, alongside other proteins that stimulate cellular immunity for the development of new vaccines to combat *E. tenella* infection in chickens.

## Conclusions

In this study, *Et*CHP18905 was amplified, expressed and characterized. Its location on Spz and Mrz was determined. Anti-r*Et*CHP18905 antibodies could reduce the rate of Spz invasion. The results of animal immune protection assays indicated that vaccination with r*Et*CHP18905 was capable of eliciting both humoral immunity and cell-mediated immunity, providing moderate protective immunity against *E. tenella*. However, the exact roles of *Et*CHP18905 in coccidial infections require further investigation.

## Competing interest

The authors declare that they have no competing interests.
